# Impacts of Hydrogen Adsorption on Carbon Nanotube–Metal Schottky Contacts

**DOI:** 10.3390/ma18061202

**Published:** 2025-03-07

**Authors:** Chuntian Huang, Nini Ye, Haijun Luo, Hezhu Shao, Weijin Qian, Chaolong Fang, Changkun Dong

**Affiliations:** Wenzhou Key Lab of Micro-Nano Optoelectronic Devices, Wenzhou University, Wenzhou 325035, China; chunthuang@outlook.com (C.H.); 15284690458@163.com (N.Y.); luohaijun@wzu.edu.cn (H.L.); hzshao@wzu.edu.cn (H.S.); weijinqian@wzu.edu.cn (W.Q.); fansy21@163.com (C.F.)

**Keywords:** carbon nanotubes, Schottky contact, gas sensing, hydrogen, work function

## Abstract

Carbon nanotube (CNT)–metal Schottky contacts are widely employed in different types of electronic devices, including field effect transistors (FET) and gas sensors. CNTs are normally considered stable on electronic properties with gas adsorptions. In this work, performance changes of the multi-walled carbon nanotube (MWCNT)–metal junctions related to hydrogen adsorptions were illustrated. MWCNT/Pd and MWCNT/Au Schottky junctions based resistive sensors were constructed to investigate the low-pressure gas sensing performances for hydrogen in the range of 10^−6^~10^−3^ Pa. Two types of sensors presented opposite behaviors with hydrogen adsorptions, i.e., the sensor resistance rose for the MWCNT/Pd sensor but dropped for the MWCNT/Au sensor with increasing hydrogen pressure. The work function reductions of Pd and CNT are considered the key cause, which could change the Schottky barrier properties dramatically. This behavior may play crucial roles for the accurate utilization of CNT-based Schottky devices.

## 1. Introduction

Hydrogen energy is rapidly being developed as a green source to meet the challenges of climate change and energy sustainability [1,2]. Hydrogen gas is colorless, odorless, and flammable, thus may bring about safety issues, especially when a leak occurs without effective detection [3]. Therefore, there is wide demand for various types of hydrogen sensors, including low-pressure hydrogen gas sensors. Various nanomaterials and their compounds have been used for hydrogen sensing, such as noble metals, metal oxide semiconductors, Xenes, and palladium films [4,5]. Reliable hydrogen sensing techniques are crucial for safe applications of hydrogen energies in the fields of fuel cells and renewable energy sources [6,7]. Schottky devices formed by connecting metals with semiconductors are widely used in gas sensing, integrated circuits, and other fields [8,9].

Carbon nanotubes (CNTs) possess superior physical and structural properties, and Schottky devices based on CNTs show excellent performance for applications [10,11,12,13,14] with notable characteristics including safety, miniaturization, and energy efficiency. In a CNT field effect transistor (FET), the carbon nanotube, as the conductive channel, connects the metal source and drain to form Schottky contacts, which regulate the number of carriers through the gate to realize switching characteristics. For gas sensing, the gas adsorption changes the work function of the metal, leading to a change in the Schottky barrier, which affects the carrier transport performance [15]. Reversible palladium hydride forms with hydrogen adsorption on Pd, which will affect physical or structural properties of Pd, including work function, conductivity, lattice constant, and optical properties [16]. A high-performance FET with a subthreshold swing of 73 mV/dec and transconductance of 1 mS/μm was fabricated employing an array of carbon nanotubes for integrated circuit devices [17]. The resistance sensor is another device based also on Schottky contact, in which CNT connects two metal electrodes, and the metal work function changes with gas adsorption, so that the carrier transport between CNT and metal changes, resulting in a change in the device resistance [18,19]. A CNT-modified resistance sensor with silver nanoparticles is able to detect acetone at room temperature [20]. An ultra-sensitive CNT hydrogen sensor reached sub-ppm detection at room temperature with a rapid response [21]. A suspended cross-stacked MWCNT sensor achieved 35.3% resistance change at 4% H_2_, enabling scalable production [22]. A sensitive and highly selective CNT-based room temperature ammonia resistance sensor was developed from the charge transfer when the MoS_2_-CNT composite contacts the reducing gas [23].

Normally, CNTs are considered stable with both structures and properties for electronic device applications. However, our studies showed that gas adsorptions, especially dissociated atomic hydrogen adsorption, could reduce CNT work functions significantly [24,25]. Therefore, it is of great significance to study the impacts of CNT electronic property changes with gas adsorptions for CNT nano-devices, including the barrier modulation and carrier transport properties for CNT Schottky devices. In this work, the MWCNT–metal Schottky contact resistance sensor was developed through photolithographic and dielectrophoresis (DEP) processing, and the electrical properties of the sensor under a hydrogen environment were studied by experimental and simulation investigations, revealing charge redistribution of the metal–MWCNT contact with the work function modulation from the gas adsorption. Two types of sensors developed from MWCNT-Pd and MWCNT-Au present distinctive hydrogen sensing performances in the low pressure range of 10^−6^ to 10^−3^ Pa with low power consumption.

## 2. Materials and Methods

### 2.1. Device Preparation

**Figure 1:**
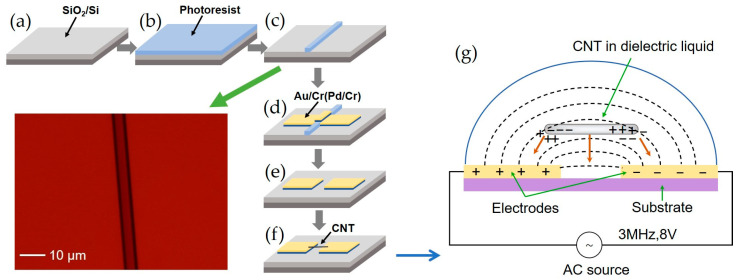
Production process of CNT sensors. (**a**) SiO_2_/Si substrate; (**b**) spin-coating photoresist layer; (**c**) the lithography process leaves a mask layer (as a subsequent sacrifice layer); (**d**) magnetron sputtering deposited metal films; (**e**) removal of the sacrificial layer and the metal above; (**f**) deposition of carbon nanotubes by DEP; (**g**) schematic of DEP assembly of CNT across electrodes.

The gas sensor was developed on a p-type silicon wafer with a thickness of 500 μm and resistivity of 0.01 Ω·cm as the substrate, on which there is a 300 nm thick silicon dioxide layer. Metal electrodes were prepared on the oxide layer by the lift-off process [26], and carbon nanotubes were deposited between the metal electrodes by the DEP process [27,28]. Figure 1 shows the production flow chart of two types of sensors based on Pd and Au, respectively. Firstly, a graphic lithographic adhesive sacrificial layer was obtained through the lithography process, as shown in Figure 1a–c. Secondly, magnetron sputtering was used to deposit a Cr adhesive layer and a metal electrode of ~100 nm in thickness from Au or Pd, to develop the MWCNT/Au sensor or MWCNT/Pd sensor, as shown in Figure 1d. After the metal film deposition, the sacrificial layer and the metal above were removed by soaking and ultrasonic processing. Then, two electrodes with a distance of 3 μm can be obtained, as shown in Figure 1e. In the final step (Figure 1f), carbon nanotubes were deposited between two metal electrodes by DEP, which is depicted in Figure 1g.

DEP deposition utilizes dielectrophoretic forces generated by a non-uniform electric field to deposit carbon nanotubes between metal electrodes. When CNTs are placed in a non-uniform electric field, their internal charge distributions are rearranged, forming dipoles. Due to their conductivity and dielectric properties, CNTs exhibit significant polarization behavior within the electric field. Non-uniform electric fields could continuously alter the direction of the electric field, thereby adjusting the polarization orientation of CNTs. By adjusting the electric field frequency, strength, and solution properties, the position and orientation of carbon nanotubes can be precisely controlled. This effectively controls the motion of CNTs, avoids electrochemical reactions and thermal effects, and enhances the uniformity and precision of deposition [29,30]. To prepare the DEP solution, MWCNTs (Aladdin, CA, USA) with lengths of 5–30 μm and diameters of 10–30 nm were dispersed uniformly in the sodium dodecyl benzene sulfonate (SDBS, SCRC, Shanghai, China) solution through ultrasonic oscillation. Then, the MWCNT solution was dripped between two metal electrodes while an AC voltage in square wave with a frequency of 3 MHz and a voltage of 8 V was applied. Finally, the MWCNT connection was constructed between two electrodes.

### 2.2. Characterization and Performance Tests

The MWCNT material and the sensor were characterized by scanning electron microscopy (SEM, JSM-7100F, JEOL, Tokyo, Japan), transmission electron microscopy (TEM, Titan G2 60–300, FEI, Hillsboro, OR, USA), Raman spectroscopy (DXR3, Thermo Scientific, Waltham, MA, USA), and X-ray photoelectron spectroscopy (XPS, Thermo Fisher Scientific escalab250×, Waltham, MA, USA).

**Figure 2:**
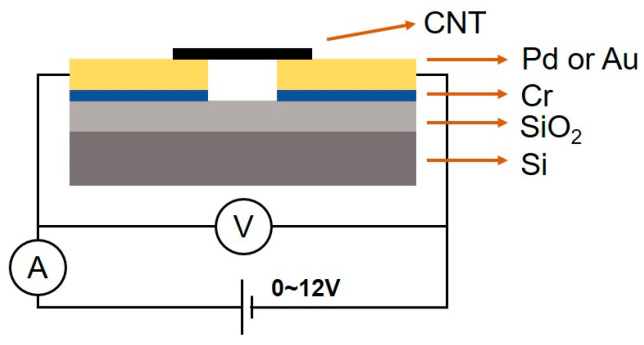
Sensor structure and test circuit.

The sensor structure and the circuit set-up are shown in Figure 2. The sensor was tested in a turbo pump high vacuum system with a background pressure of 10^−7^ Pa. The gas sensing performances of two types of structures with Au/Cr electrodes and Pd/Cr electrodes were investigated in hydrogen environments, from 10^−7^ to 10^−3^ Pa. During the test, the gas was purged into the system to a specific pressure first, then a voltage of 12 V was applied across two metal electrodes. One minute later, the voltage and current of the sensor were recorded. Subsequently, the power source was cut off and the gas purging was stopped, followed by a recovery period of one hour. During the recovery, the sensor resistance was measured every twenty minutes. Upon completion of the recovery, the gas was introduced again to a higher pressure for the next test.

The sensing properties were characterized by the current and corresponding resistance changes. The resistance changes under different gas pressures are defined as Response = (R − R_0_)/R_0_, where R_0_ is the resistance of the sensor under ultimate vacuum, and R is the resistance under the test gas pressure. ΔI, the difference between currents under the test pressure and the ultimate vacuum, is also presented.

### 2.3. The First-Principles Simulation Study

A first-principles simulation was conducted to understand the mechanisms of sensing performances with gas adsorptions on different metal electrodes. The simulation was based on the Vienna Ab initio Simulation Package (VASP, version 3.2.9) [31,32,33], and the Perdew–Burke–Enzerhoff (PBE) functional and all-electron projection enhanced wave (PAW) methods were used to deal with the exchange-correlated energies. When solving the Kohn–Sham equation for the plane wave expansion, a cut off energy of 450 eV was used.

We modeled a p (3 × 3) Pd (111) or p (3 × 3) Au (111) surface as a slab with periodic repeats of four atomic layers, while the top two layers were relaxed and the bottom two layers were fixed. To ensure that there was no interaction between the slabs, a vacuum space of 15 Å in the z-direction was introduced. The Brillouin region was sampled by (6 × 6 × 1) Monhorst-Pack mesh K-points.

## 3. Results and Discussion

### 3.1. Carbon Nanotubes and Schottky Contact

**Figure 3:**
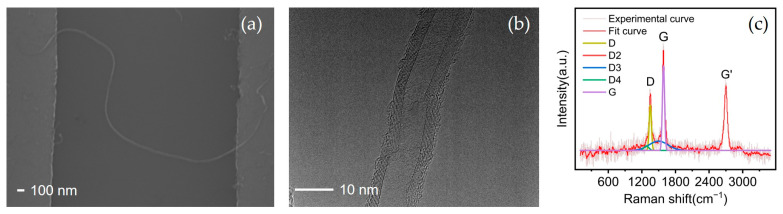
(**a**) SEM image of MWCNT–metal electrode contacts; (**b**) TEM of MWCNT; (**c**) Raman spectra of MWCNT.

The MWCNT–metal electrode contacts after DEP are shown in Figure 3a. A single MWCNT bridges two metal electrodes. A TEM image of the MWCNT is shown in Figure 3b. The CNTs are multi-walled CNTs (MWCNTs), consisting of approximately eight layers, with diameters of about 15 nm. The Raman spectrum of CNTs is characterized by three primary peaks: the D-band (~1350 cm^−1^), which is associated with defects and disorder; the G-band (~1580 cm^−1^), originating from the in-plane vibration of sp^2^-hybridized carbon atoms and indicative of graphitization; and the 2D-band (~2700 cm^−1^), a second-order mode reflecting layer stacking and electronic interactions. The intensity ratio of the D-band to the G-band (I_D_/I_G_) serves as a key metric for the defect density, while the shape and intensity of the 2D-band provide insights into the number of layers and interlayer coupling. The I_D_/I_G_ ratio is calculated based on the areas under the D and G Raman peaks from deconvoluted Raman spectra [34]. Together, these features enable comprehensive characterization of CNTs, including defect analysis, layer number estimation, and evaluation of stress or doping effects [35]. Figure 3c shows the Raman spectra of CNTs with a D-peak of about 1343 cm^−1^ and G-peak of about 1581 cm^−1^. The I_D_/I_G_ ratio of 0.55 indicates good crystallinity of the MWCNT structure.

**Figure 4:**
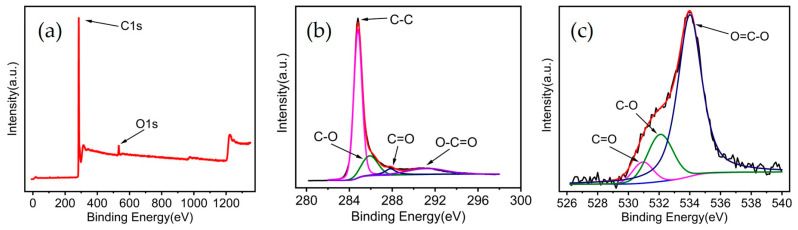
XPS spectra of MWCNT. (**a**) survey; (**b**) C 1 s; (**c**) O 1 s.

XPS characteristics were further investigated for MWCNTs, as shown in Figure 4a. For C 1 s spectra (Figure 4b), a major peak from C-C was observed at 284.8 eV. In addition, two minor peaks from C-O and C=O bonds could be seen at 285.9 eV and 287.8 eV, respectively [36]. For O 1 s spectra (Figure 4c), two peaks at 531 eV and 532.1 eV were C=O and C-O bonds, respectively [37]. The appearance of an oxygen peak is associated with absorbed water molecules and air adsorption [38,39].

### 3.2. Hydrogen Sensing Performances

**Figure 5:**
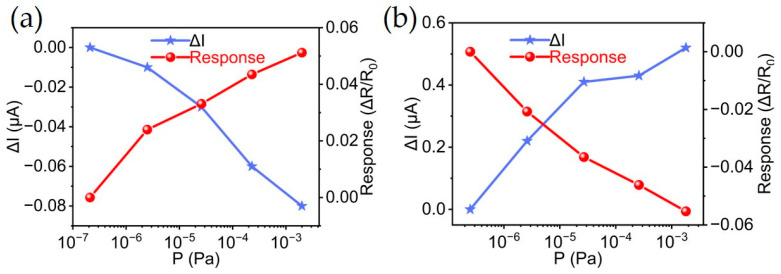
Current and resistance performances in hydrogen ambience of 10^−7^ to 10^−3^ Pa for (**a**) MWCNT/Pd sensor and (**b**) MWCNT/Au sensor.

Two types of sensors were tested in hydrogen environments. For the MWCNT/Pd sensor, the sensor current decreased by about 0.08 μA as the hydrogen pressure increased across six orders of magnitude, from 10^−7^ to 10^−3^ Pa, as shown in Figure 5a. This corresponds to a substantial current reduction, indicating a sensitivity to hydrogen pressure changes. Concurrently, the sensor’s resistance exhibited a corresponding increase with a change rate of 5.12%, demonstrating an inverse relationship with the current. On the contrary, the MWCNT/Au sensor exhibited a current increase of approximately 0.52 μA as the hydrogen pressure rose from 10^−6^ to 10^−3^ Pa, accompanied by a resistance drop with a change rate of 5.53% (Figure 5b). This behavior reveals an opposite phenomenon compared to the MWCNT/Pd sensor.

### 3.3. Mechanism of Hydrogen Adsorption

**Figure 6:**
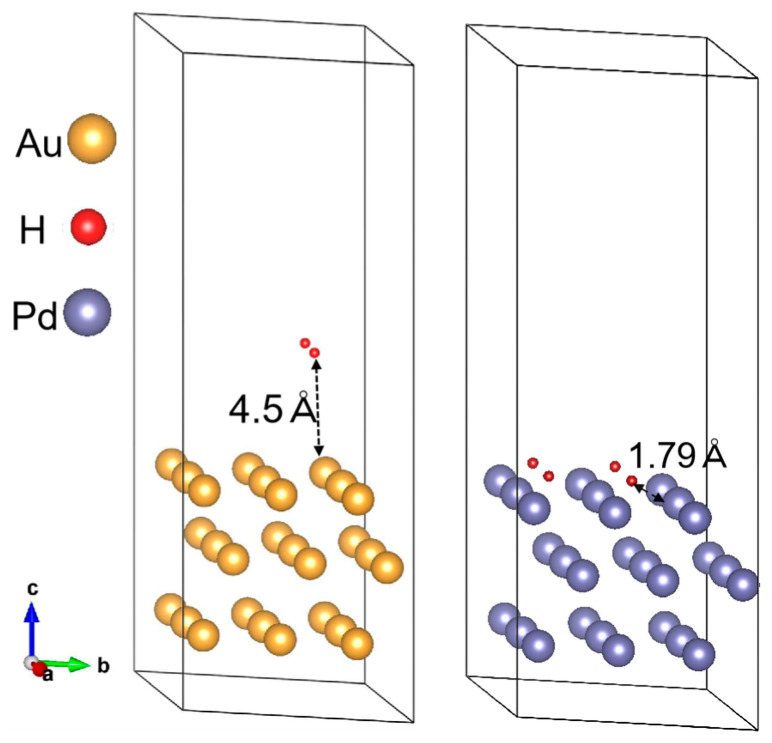
H_2_ on Au (111) and atomic H on Pd (111) surface.

For the MWCNT/Pd sensor in hydrogen ambience, hydrogen molecules would dissociate on the palladium surface, and our simulation shows an intermediate state with bond elongation from 0.73 Å to 0.89 Å. This dissociative adsorption process results in the subsequent dissolution of atomic hydrogen into the Pd substrate with a H-Pd bond length of 1.79 Å (Figure 6), which induces a measurable reduction in the work function of the palladium surface [40,41]. Halas et al. have demonstrated that hydrogen adsorption could reduce the work function of Pd by 2.0 eV [42]. However, for the MWCNT/Au sensor, calculations showed Au lacks reactivity with hydrogen because Au has both a very high barrier and the least stable chemisorption state [15,43,44], which makes Au form a weak bond with H. Our simulation shows the hydrogen molecule is about 4.5 Å away from the Au (111) surface, as shown in Figure 6. Therefore, the work function of Au does not change significantly upon hydrogen adsorption.

During the operation of the sensor, hydrogen would also dissociate on CNT, initiated by the Joule energy input under the microampere current through the CNT [45,46,47]. Our previous first-principles calculations revealed that the incorporation of hydrogen atoms could reduce the work function of CNTs [24,48], and experimental measurements of the field emission electron energy distribution demonstrated a reduction of 0.36 eV in the work function of MWCNTs upon hydrogen adsorption [48].

**Figure 7:**
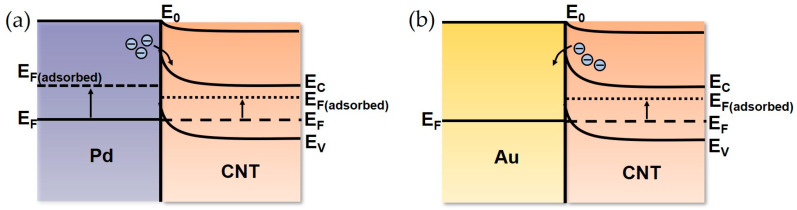
Energy band diagrams of the electrode–MWCNT Schottky contacts under hydrogen adsorptions for (**a**) MWCNT/Pd sensor and (**b**) MWCNT/Au sensor.

The influence of work function variations could be revealed from the energy band diagram. For intrinsic materials, the work function of Pd is higher than that of CNT, and the energy band diagram is shown in Figure 7a for the Pd-MWCNT contact. After the contact, electrons move from higher energy levels to lower energy levels, specifically from CNT to Pd, resulting in the formation of the hole inversion layer and upward bending of the energy band. As a result, both internal and surface electron energy levels experience alteration until Fermi levels reach the same level. After hydrogen adsorption with the formation of a PdH_x_ compound [49], it is expected that the work function of Pd drops more than that of CNT, leading to a higher Fermi level. Consequently, electrons transfer from Pd to CNT, which is opposite to the initial state. CNTs generally possess p-type behavior [49,50,51]. Thus, the capture of electrons would fill the conductive holes, causing the resistance to increase [49]. As for the Au-MWCNT contact, the work function reduction of CNT after hydrogen adsorption makes its Fermi energy level higher than Au, resulting in the electron flow from CNT to Au, as shown in Figure 7b. As a result, the hole concentration in CNT increases and the resistance declines.

### 3.4. Recovery After Hydrogen Adsorption

**Figure 8:**
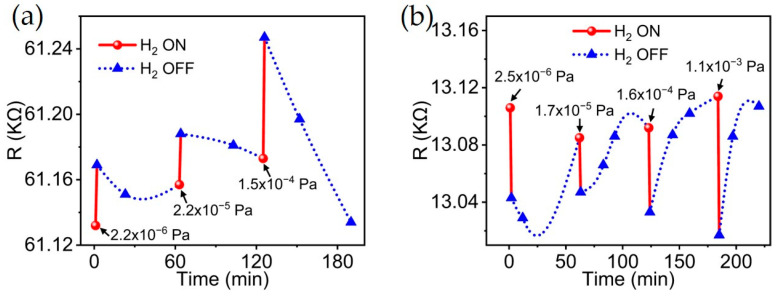
The hydrogen sensing responses of (**a**) MWCNT/Pd sensor and (**b**) MWCNT/Au sensor.

The sensing and recovery responses of the MWCNT/Pd sensor under a hydrogen atmosphere of 10^−6^ to 10^−4^ Pa were investigated, as shown in Figure 8a. With the increase in hydrogen pressure, the conductivity of the sensor decreased and the resistance increased. After stopping the ventilation, the resistance recovered by about 30~50% in one hour at 10^−6^ Pa and 10^−5^ Pa, and the recovery sped up with increasing pressure. For the MWCNT/Au sensor, as shown in Figure 8b, the resistance showed a trend of decreasing with the increase in hydrogen pressure above 10^−5^ Pa, and the resistance recovered by about 70~95% within one hour after terminating the hydrogen. For the MWCNT/Pd sensor, hydrogen would desorb from both the CNT and Pd electrode during the recovery. H atoms opt to permeate into Pd [52], resulting in the slow desorption and recovery.

## 4. Conclusions

In summary, MWCNT/Pd and MWCNT/Au Schottky junction-based resistive gas sensors were constructed to study the low-pressure gas sensing performances for hydrogen gas. Performance changes of the MWCNT–metal junctions related to hydrogen adsorptions were demonstrated. With increasing gas pressure from 10^−7^ to 10^−3^ Pa, two types of sensors exhibited opposite behaviors in hydrogen ambience. The resistance of the MWCNT/Pd sensor rose, but the resistance declined for the MWCNT/Au sensor, attributed to different work function behaviors for Pd, Au, and MWCNT. With H_2_ adsorptions, the consequent Schottky barrier changes would be different for two sensors, because the work function of Pd decreases more than that of CNT, resulting in the electron transport from Pd to CNT. While Au is inert to hydrogen, the electron would transport from CNT to Au. This behavior should be taken into account for the development of CNT-based Schottky devices. The quicker recovery of the MWCNT/Au sensor implies that hydrogen desorption would be easier from CNTs than from Pd.

## Data Availability

The original contributions presented in the study are included in the article, further inquiries can be directed to the corresponding author.
